# Pooled prevalence and determinants of skilled birth attendant delivery in East Africa countries: a multilevel analysis of Demographic and Health Surveys

**DOI:** 10.1186/s13052-020-00943-z

**Published:** 2020-11-30

**Authors:** Zemenu Tadesse Tessema, Getayeneh Antehunegn Tesema

**Affiliations:** Department of Epidemiology and Biostatistics, Institute of Public Health, College of Medicine and Health Sciences, University of Gondar, Gondar, Ethiopia

**Keywords:** Skilled birth attendant, East Africa, Pooled analysis, DHS, Multilevel analysis

## Abstract

**Introduction:**

Skilled health professional assisted delivery is an effective strategy to reduce maternal and newborn mortality. Skilled assistant delivery can prevent about 16–33% of maternal and newborn mortality. Despite the commitments of the government to assure home free delivery, majority of the births in Sub-Saharan Africa are attended by traditional birth attendants. As to our search of the literature, there is limited evidence on the prevalence and determinants of skilled delivery in East African countries. Therefore, this study aimed to estimate the pooled prevalence and determinants of skilled birth attendant delivery in East Africa Countries.

**Methods:**

Pooled analysis was done based on Demographic and Health Surveys conducted in the 12 East African countries from 2008 to 2017. A total weighted sample of 141,483 women who gave birth during the study period was included in the study. The pooled prevalence of skilled birth attendance was estimated using STATA version 14. Intra-class Correlation Coefficient, Median Odds Ratio, Proportional Change in Variance, and deviance were used for model fitness and comparison. The multilevel multivariable logistic regression model was fitted to identify determinants of skilled birth attendance in the region. Adjusted Odds Ratio with its 95% Confidence Interval was used to declare significant determinants of skilled birth attendants.

**Results:**

The pooled prevalence of skilled birth attendant in East African countries were 67.18% (95% CI:66.98, 67.38) with highest skilled birth attendant in Rwanda (90.68%) and the lowest skilled birth attendant in Tanzania (11.91%). In the Multilevel multivariable logistic regression model; age 15–24 (Adjusted Odds Ratio (AOR) = 1.14, 95%CI:1.09, 1.18), age 25–49(AOR = 1.16, 95%CI:1.10,1.23), primary women education (AOR = 1.57, 95%CI:1.51,1.63), secondary and above women education (AOR = 2.85, 95%CI:1.73,3.01), primary husband education (AOR = 1.11, 95%CI = 1.07,1.15), secondary and above husband education (AOR = 1.46, 95%CI = 1.40,1.53), middle wealth index (AOR = 1.43, 95%CI = 1.38,1.49),rich wealth index (AOR = 2.38, 95%CI = 2.28,2.48), had ANC visit (AOR = 1.68, 95%CI = 1.62,1.73),multiple gestation (AOR = 2.06, 95%CI = 1.90,2.25), parity 2–4(AOR = 0.65, 95%CI = 0.61,0.69), parity 5 + (AOR = 0.44, 95%CI = 0.41,0.47), accessing health care not big problem (AOR = 1.32, 95%CI = 1.28,1.36), residence (AOR = 0.43, 95%CI = 0.41,0.45) and being Burundi resident (AOR = 0.77, 95%CI = 0.70,0.85) were significantly associated with skilled assisted delivery.

**Conclusion:**

Skilled birth attendance at birth in the East Africa countries was low. Maternal age, women and husband education, wealth index, antenatal care visit, multiple gestations, parity, accessing health care, residence, and living countries were major determinants of skilled attendant delivery. Strategies to increase the accessibility and availability of healthcare services, and financial support that targets mothers from poor households and rural residents to use health services will be beneficial. Health education targeting mothers and their partner with no education are vital to increasing their awareness about the importance of skilled birth attendance at birth.

## Introduction

Every day, about 830 women die from preventable causes related to pregnancy and childbirth, of which 99% of all maternal deaths occur in developing countries [1]. Worldwide, maternal mortality fell from 385 deaths per 100,000 livebirths in 1990 to 216 deaths per 100,000 livebirths in 2015, which is declined by 44% [2, 3]. Sustainable Development Goal (SDG) goal 3 target 3.1 calls for reduction of maternal mortality ratio less than 70 per 100,000 live births between 2016 to 2030 [4]. Worldwide, 34% of deliveries have no skilled birth attendant. That is 45 million birth occurred at home without skilled birth health personnel each year [5]. Sub-Saharan Africa has also shown progress over the same period on skilled birth attendance, and by 2012–2017 over 50% of births were attended by skilled health personnel [6].

Skilled Birth Attendant (SBA) offers a safety and life-sustaining environment for both mothers and newborns thereby reducing the chance of complications [2]. Although it has difficult to establish a causal relationship between skilled birth attendance and maternal mortality, estimates suggested that skilled birth attendants present at birth could prevent around 16 to 33% of maternal death [7].

The East African countries that include Burundi, Ethiopia, Comoros, Uganda, Rwanda, Tanzania, Mozambique, Madagascar, Zimbabwe, Kenya, Zambia, and Malawi are among the world’s poor countries with poor accessibility and affordability of maternal health care services [8].

Different scholars studied skill birth attendants at a specific population in different parts of East African Country. Studies conducted in Zambia reported that at least one ANC visit and poor economic status of respondents were associated with SBA [9]. Other scholars from Ethiopia evidenced that place residence, maternal education, distance to a health facility, the decision on place of delivery, and knowledge on obstetric danger signs after delivery was associated with SBA [10]. Educational status [10–14], antenatal care visit [15–18], age group [19], previous pregnancy complication [10, 14, 20], decision of place of delivery [14], parity [20], husband education [12], wealth index [11, 19, 21, 22], accessing health care [18], sex of provider [23], residence [22], and maternal occupation [20] were identified as significant factors associated with SBA.

Despite the implementation of intensive public health interventions to skilled assisted delivery, the majority of the birth in SSA particularly in East African countries are attained by traditional birth attendants and continued to share the largest portion of global maternal, and newborn mortality. As to our search of literature, there is limited evidence on the magnitude and factors associated with skilled assistant delivery in East African countries.

Therefore, this study aimed to estimate the pooled prevalence and determinants of skilled birth attendance in the 12 East Africa Countries from 2008 to 2017 using recent Demographic and Health Surveys. This could help to design evidence-based public health decisions for enhancing skilled birth attendance at birth to reduce maternal and newborn mortality in these countries.

## Methods

### Data source, tool, and sampling procedure

**Fig. 1:**
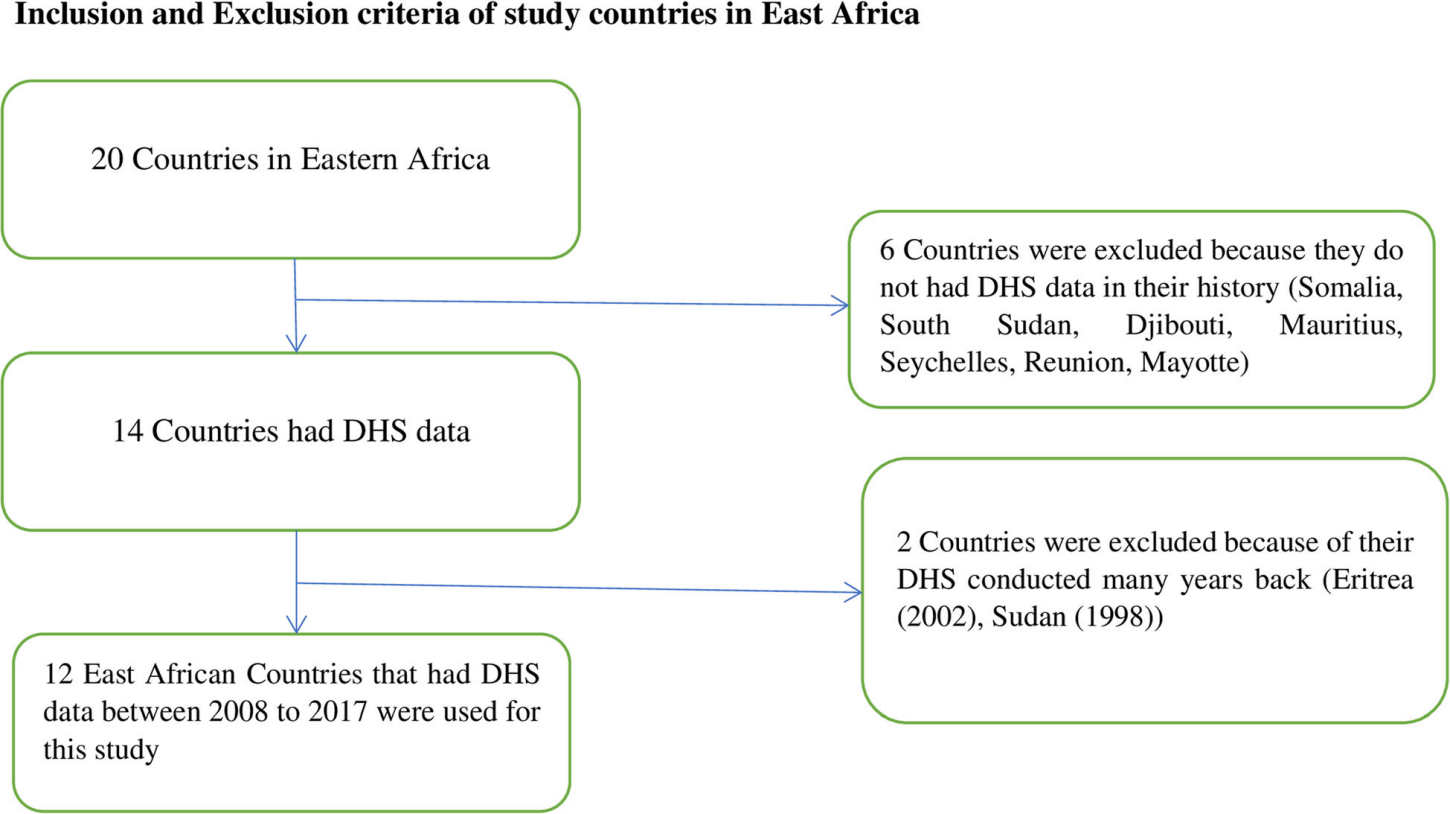
Schematic diagram of selection of study countries among East African countries

The data was obtained from the measure DHS program at www.measuredhs.com after prepared concept notes about the project. The Demographic and Health Survey (DHS) data were pooled from the 12 East Africa Countries from 2008 to 2017. The recent DHS of Country-specific dataset was extracted during the specified period. The 12 East Africa Countries in which data extracted include Burundi, Ethiopia, Kenya, Comoros, Madagascar, Malawi, Mozambique, Rwanda, Tanzania, Uganda, Zambia, and Zimbabwe (Table 1). There 20 countries in WHO regions of East Africa. In history, only 14 countries had DHS data. For this study 12 countries were included (Fig. 1). The DHS program adopts standardized methods involving uniform questionnaires, manuals, and field procedures to gather the information that is comparable across countries in the world. DHSs are nationally representative household surveys that provide data from a wide range of monitoring and impact evaluation indicators in the area of population, health, and nutrition with face to face interviews of women age 15 to 49. The surveys employ a stratified, multi-stage, random sampling design. Information was obtained from eligible women aged 15 to 49 years in each country. Detailed survey methodology and sampling methods used in gathering the data have been reported elsewhere [24].

**Table 1:** The DHS years of study and study participants of the skilled birth attendant in the 12 East African Countries from 2008 to 2017

Eastern Region of Arica	DHS year	Study participants
Burundi	2010	13,610
Ethiopia	2016	11,022
Kenya	2014	19,563
Comoros	2012	2880
Madagascar	2008/09	12,407
Malawi	2015/16	17,395
Mozambique	2011	11,477
Rwanda	2014/15	10,051
Tanzania	2015/16	8002
Uganda	2011	15,270
Zambia	2017	13,383
Zimbabwe	2013/14	6418
Total sample size		**141,483**

### Variables

#### Outcome variable

The response (outcome) variable of this study was a skilled birth attendant. The response variable was generated from the question asked to the women who gave birth within 5 years preceding the survey question “who assisted the delivery?” The response was dichotomized as a health professional and another person. Health professionals include doctors, nurses, nurse/midwife, auxiliary midwife, and others (health officer and health extension workers). Other persons include traditional birth attendance (TBA), traditional health volunteer, community/village health volunteer, neighbors/friends, relatives, others. If a women delivery were assisted by health professional coded as “1”, otherwise coded as “0”.

#### Independent variables

Based on the literature, the independent variables included in this were two types of variables. Individual-level and community-level variables. Community-level variables include country and residence. The individual-level variables are age group, marital status, maternal and husband educational status, occupational status, wealth index, parity, ANC visit, wanted pregnancy, number of gestation, accessing health care wealth index, and birth interval.

### Operational definition

**Accessing health care:** most studies have isolated the travel time and transport cost when looking at access to health facilities. In the DHS data, women were asked whether a range of factors would be a big problem for them in accessing health care. We generated a composite variable using each country DHS standard questions. The questions included:

getting the money needed for treatment (big problem/not a big problem)
distance to a health care facility (big problem/not a big problem)
having to take transport (big problem/not a big problem)
Not wanting to go alone (big problem/not a big problem)

If women face at least one or more of the problems (money, distance, companionship, and permission) we considered as there is health care accessing problem that was our primary interest and we coded as 1 and If they reported no health care accessing out of four (money, distance, companionship, and permission) we code 0.

#### Wealth index

Wealth index is calculated by using principal components analysis (PCA) that involves assigning scores on the indicator variables. In the dataset, the index has five quintiles such as; the lowest quintile (poorest), 2nd quintile (poorer), 3rd quintile (middle), 4th quintile (wealthier), and the 5th quintile (wealthiest). In this study for ease of analysis this variable was recategorized as ‘poorest’ and ‘poorer’ were coded as (1) ‘poor’, the middle was coded as (2) ‘middle’, and ‘wealthier’ and ‘wealthiest’ were coded as (3) ‘rich” [25].

**Auxiliary midwife:**-is a village-level female health worker who is known as the first contact.

### Data management and analysis

The data was cleaned by STATA version 14.1 software. Sample weighting was done for further analysis.

### Multi-level analysis

Since the outcome variable was binary two-level mixed-effects logistic regression analysis was employed. Sampling weight was applied as part of a complex survey design using primary sampling unit, strata, and women’s individual weight (V005).

The individual and community level variables associated with skilled birth attendant were checked independently in the bi-variable multilevel logistic regression model and variables which were statistically significant at *p*-value 0.20 in the bi-variable multilevel mixed-effects logistic regression analysis were considered for the final individual and community level model adjustments. In the multivariable multilevel analysis, variables with a *p*-value≤0.05 were declared as significant determinants of skilled assistance delivery.

### Model building

Four models were fitted. The first was the null model containing no exposure variables which was used to check variation in community and provide evidence to assess random effects at the community level. Then model I was the multivariable model adjustment for individual-level variables and model II was adjusted for community-level factors. In model III, possible candidate variables from both individual and community-level variables were fitted with the outcome variable.

### Parameter estimation method

The fixed effects (a measure of association) were used to estimate the association between the likelihood of skilled birth attendant and explanatory variables at both community and individual level and were expressed as odds ratio with 95% confidence interval. Regarding the measures of variation (random-effects), Community-level variance with standard deviation, intracluster correlation coefficient (ICC), Proportional Change in Community Variance (PCV), and median odds ratio (MOR) was used.

The aim of the median odds ratio (MOR) is to translate the area level variance in the widely used odds ratio (OR) scale, which has a consistent and intuitive interpretation. The MOR is defined as the median value of the odds ratio between the area at the highest risk and the area at the lowest risk when randomly picking out two areas. The MOR can be conceptualized as the increased risk that (in median) would have if moving to another area with a higher risk.

It is computed by; MOR = exp[√(2×Va)×0.6745] [26].

Where; VA is the area level variance, and 0.6745 is the 75th centile of the cumulative distribution function of the normal distribution with mean 0 and variance 1. See elsewhere for a more detailed explanation [24]. Whereas the proportional change in variance is calculated as [27]

$$ \mathrm{PCV}=\left[\left(\mathrm{VA}-\mathrm{VB}\right)/\mathrm{VA}\right]\ast 100; $$

Where; where VA = variance of the initial model, and VB = variance of the model with more terms.

## Results

### Socio-demographic characteristics

A total of 141,483 women who gave birth in the 5 years preceding each country’s DHS survey were included in this study. The median age of women was 28 with IQR of 10 with the majority of women underlie in the age group of 25–34. Most of the women were from Kenya 19,563(13.83%) and the smallest number of women were included from Comoros 2880(2.04%). Majority 110,471(78.08%) of women were from rural residents. A high number of women 100,261 (70.86%) were married. Two out of three women had antenatal care visit 93,360(65%). A large number of 80,769(57.09%) responded that they faced accessing health care service problems (Table 2).

**Table 2:** Socio-demographic, economic, maternal and obstetric characteristic respondents in the 12 East Africa Countries from 2008 to 2017

Variables	Weighted Frequency (***N*** = 141,483)	Percentage (%)
**Socio-economic and demographic characteristics of respondents**		
**Country**		
Burundi	13,610	9.62
Ethiopia	11,022	7.79
Kenya	19,563	13.83
Comoros	2880	2.04
Madagascar	12,407	8.77
Malawi	17,395	12.29
Mozambique	11,477	8.11
Rwanda	8002	5.66
Tanzania	10,051	7.10
Uganda	15,270	10.79
Zambia	13,383	9.46
Zimbabwe	6418	4.54
**Residence**		
Urban	31,012	21.92
Rural	110,471	78.08
**Age group**		
15–24	42,166	29.80
25–34	67,704	47.85
35–49	31,612	22.34
**Marital status**		
Single	41,222	29.14
Married	100,261	70.86
**Maternal education**		
No education	33,631	23.77
Primary	75,945	53.68
Secondary and above	31,906	22.55
**Husband education**		
No education	48,411	34.22
Primary	59,332	41.94
Secondary and above	33,739	23.85
**Maternal Occupation**		
Had no Occupation	47,153	33.33
Had Occupation	94,330	66.67
**Wealth Index**		
Poor	64,367	45.49
Middle	27,586	19.50
Rich	49,528	35.01
**Maternal and Obstetrics characteristics of respondents**		
**ANC visit**		
No ANC visit	48,122	34.01
Had ANC visit	93,360	65.99
**Wanted Pregnancy**		
Yes	121,189	85.66
No	20,294	14.34
**Number of gestation**		
Single	136,979	96.82
Multiple	4054	3.18
**Parity**		
1	22,141	15.65
2–4	72,857	51.50
5+	46,848	32.86
**Accessing health Care**		
Big problem	80,769	57.09
Not bog problem	60,714	42.91
**Birth Interval**		
Less than 24 month	52,549	37.14
24 month and above	88,934	62.86

### Pooled prevalence of skilled birth attendant

**Fig. 2:**
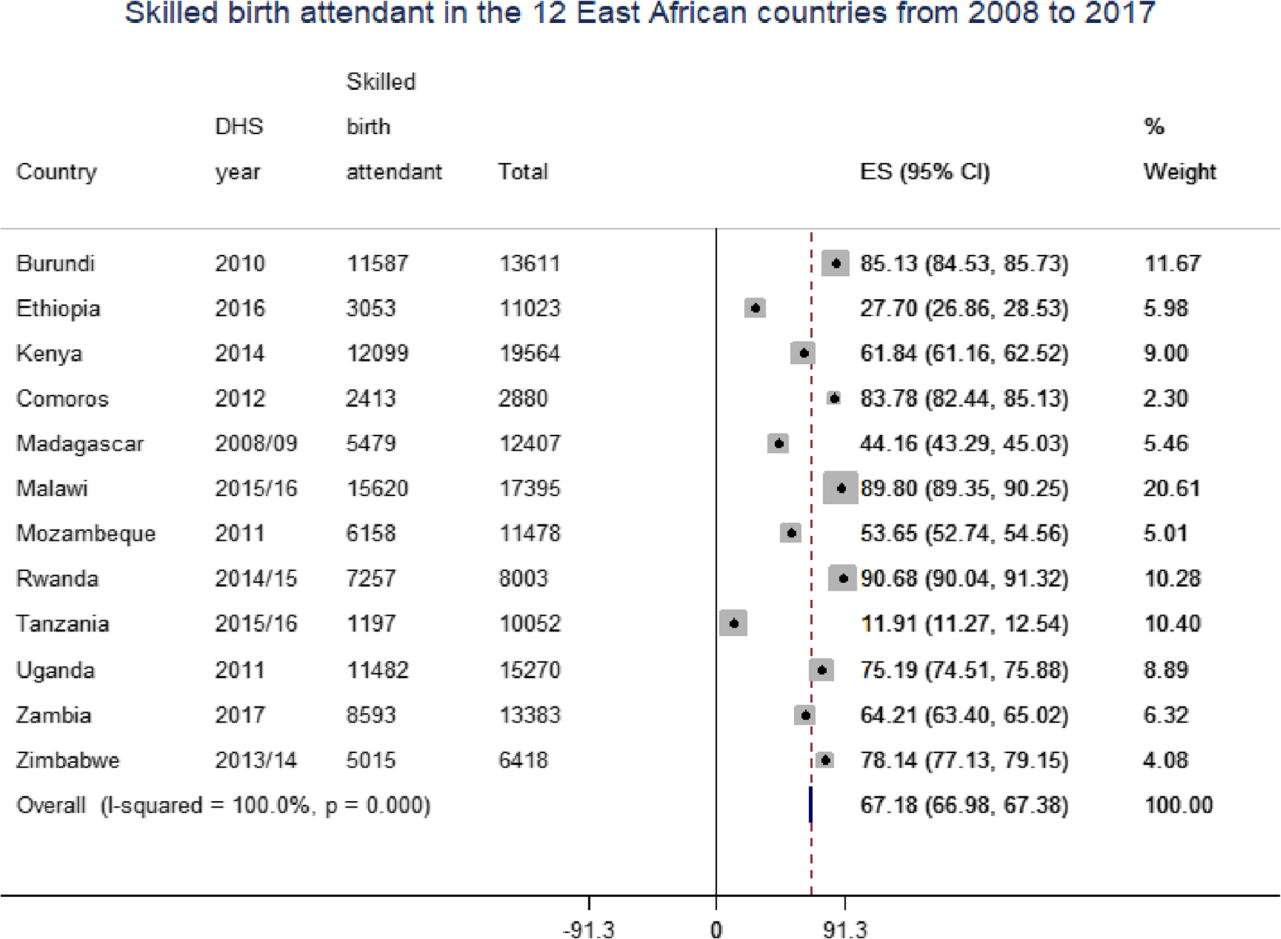
Forest plot of the skilled birth attendant in East African Country from 2008 to 2017

The pooled prevalence of skilled birth attendants in East African countries was 67.18% (95% CI: 66.98, 67.38) with the highest skilled birth attendant in Rwanda (90.68%) and the lowest skilled birth attendant in Tanzania (11.91%) (Fig. 2).

### Determinants of skill birth attendant at birth in East Africa countries

#### The random-effects analysis results

The fixed effects (a measure of association) and the random intercepts for skilled birth attendants are presented in Table 2. The results of the empty model revealed that there was statistically significant variability in the odds of seeking skilled birth attendance with community variance (τ = 0.79, *p*-value = *p* < 0.001). Similarly, the ICC in the empty model implied that 19.39% of the total variability in skilled assistant delivery was attributed to the differences between communities. Moreover, the MOR was 2.32 (95% CI 2.22,2.42) which implied that the odds of giving birth by the skilled birth attendant were 2.32 times higher when respondents moved from low to high-risk communities. This showed that the existence of significant heterogeneity in skilled assistant delivery across different communities. In the full model (model adjusted for both individual and community-level factors) community variance (community variance = 0.075; SE 0.0046; *P*-value, < 0.001), remained significant but reduced. About 7.50% of the total variance of skilled birth attendant delivery can be attributed to the contextual-level factors that remained significant even after considering some contextual risk factors. The proportional change in variance (PCV) in this model was 90.50% which showed that 90.50% of community variance observed in the null model was explained by both community and individual level variables (Table 3).

**Table 3:** Multivariable multilevel logistic regression analysis of both individual and community-level factors associated with skilled birth attendant delivery in East Africa countries from 2008 to 2017

Individual and community-level variables	Models			
	Null model	Model I	Model II	Model III
	AOR (95%CI)	AOR (95%CI)	AOR (95%CI)	AOR (95%CI)
**Age group**				
15–24		1		1
25–34		1.19(1.15,1.24)		1.14(1.09,1.18)*
35–49		1.21(1.15,1.27)		1.16(1.10,1.23)*
**Marital status**				
Single		1		1
Married		0.86(0.83,0.88)		1.04(0.99,1.07)
**Maternal education**				
No education		1		1
Primary		1.60(1.55,1.65)		1.57(1.51,1.63)*
Secondary and above		2.65(2.54,2.77)		2.85(2.70,3.01)*
**Husband education**				
No education		1		1
Primary		1.16(1.13,1.20)		1.11(1.07,1.15)*
Secondary and above		1.63(1.57,1.69)		1.46(1.40,1.53)*
**Maternal Occupation**				
Had no Occupation		1		1
Had Occupation		1.19(1.16,1.22)		0.95(0.92,1.02)
**Wealth Index**				
Poor		1		
Middle		1.40(1.36,1.45)		1.43(1.38,1.49)*
Rich		2.14(2.33,2.49)		2.38(2.28,2.48)*
**ANC visit**				
No ANC visit		1		1
Had ANC visit		1.70(1.66,1.75)		1.68(1.62,1.73)*
**Wanted Pregnancy**				
Yes		1		1
No		1.14(1.10,1.19)		0.91(0.87,1.03)
**Number of gestation**				
Single		1		1
Multiple		1.92(1.78,2.06)		2.06(1.90,2.25)*
**Parity**				
1		1		1
2–4		0.73(0.70,0.77)		0.65(0.61,0.69)*
5+		0.47(0.44,0.50)		0.44(0.41,0.47)*
**Accessing health Care**				
Big problem		1		1
Not bog problem		1.14(1.10,1.19)		1.32(1.28,1.36)*
**Birth Interval**				
Less than 24 month		1		1
24 month and above		0.96(0.93,0.99)		0.91(0.87,0.94)*
**Residence**				
Urban			1	1
Rural			0.18(0.17,0.19)	0.43(0.41,0.45)*
**Country**				
Burundi			0.58(0.53.0.64)	0.77(0.70,0.85)*
Ethiopia			0.039(0.036,0.043)	0.05(0.04,0.06)*
Kenya			0.09(0.08,0.10)	0.09(0.085,0.104)*
Comoros			0.37(0.33,0.43)	0.55(0.47,0.63)*
Madagascar			0.37(0.33,0.43)	0.065(0.05,0.071)*
Malawi			0.07(0.06,0.078)	1.01(0.91,1.12)
Mozambique			1.11(1.01,1.23)	0.12(0.11,0.13)*
Rwanda			1	1
Tanzania			0.006(0.0062,.007) 0)	0.005(0.004,0.0057)*
Uganda			0.27(0.25,0.29)	0.27(0.25,0.30)*
Zambia			0.13(0.11,0.14)	0.12(0.11,0.13)*
Zimbabwe			0.33(0.30,0.37)	0.19(0.17,0.21)*
**Random effects**				
Community variance(SE)	**0.79(0.04)1)**	**0.446(0.026)**	**0.52(0.03)**	**0.075(0.0046)**
ICC%	**19.39%**	**11.94%**	**13.71**	**7.50%**
PCV%	**1**	**43.54%**	**34.17**	**90.50%**
MOR	**2.32(2.22,2.42))**	**1.88(1.80,1.95)**	**1.98(1.90,2.06)**	**1.63(1.58,1.69)**
**Model comparison**				
Log-likelihood ratio	**−89,568**	**−80,035**	**−68,848**	**−62,929**
Deviance	**179,136**	**160,070**	**137,696**	**125,858**

NB: * = Significant at *P*-value < 0.05 ICC = Intraclass Correlation Coefficient, *MOR* Median Odds Ratio, *PCV* Proportional Change in Variance, *SE* Standard Error, *AOR* Adjusted Odds Ratio, *ANC* Antenatal Care

#### The fixed effects analysis result

The model with smaller deviance and the largest likelihood (model IV) was best fit the data and the interpretation of the fixed effects was based on this model. Model four was adjusted for both individual and community-level factors. Consequently, respondent’s age group, maternal education, husband education, wealth index, ANC visit, multiple gestations, parity, accessing health care, birth interval, residence, and living Country were significantly associated with the skilled birth interval in the East Africa Counties. The odds of SBA delivery among women aged 25–34 and 35–49 years were increased by 14 and 16% as compared to women age group 15–24(AOR = 1.14, 95%CI = 1.09,1.18) and (AOR = 1.16, 95%CI = 1.10,1.23) respectively. The odds of obtaining SBA among women of primary and secondary and above educational level were 1.57 and 2.85 times higher as compared to women with no education (AOR = 1.57, 95%CI = 1.51,1.63) and (AOR = 2.85, 95%CI = 2.70,3.01) respectively. The odds of obtaining SBA among women of their husband’s educational level primary and secondary and above educational level were increased by 11 and 46% as compared to women with no education (AOR = 1.11, 95%CI = 1.07,1.15) and (AOR = 1.46, 95%CI = 1.40,1.53) respectively. The odds of seeking SBA among women wealth status middle and rich were 1.43 and 2.38 times higher as compared to poor women (AOR = 1.43, 95%CI = 1.38,1.49) and (AOR = 2.38, 95%CI = 2.28,2.48) respectively. The odds of seeking SBA among women who had ANC visits were increased by 68% as compared to women who had no ANC visit (AOR = 1.68, 95%CI = 1.62,1.73). The odds of seeking SBA among women who faced multiple births were 2.06 times higher as compared to women who faced single birth (AOR = 2.06, 95%CI = 1.96,2.25). The odds of obtaining SBA among women of para 2–4 and para 5+ were decreased by 35 and 56% as compared to para1(AOR = 0.65, 95%CI = 0.61,0.69) and (AOR = 0.44, 95%CI = 0.41,0.47) respectively. The odds of obtaining SBA among women of not a big problem in accessing health care were increased by 32% as compared to women of big problems in accessing health care (AOR = 0.1.32, 95%CI = 1.28,1.36). The odds of obtaining SBA among women deliver with a birth interval greater than 2 and above years were decrease by 9% as compared to women deliver with less than two-year birth spacing (AOR = 0.91, 95%CI = 0.87,0.94). The odds of seeking SBA among women of the rural resident were decreased by 57% as compared to urban women (AOR = 0.43, 95%CI = 0.41, 0.45). Living countries had a significant effect on the likelihood of obtaining SBA in the East Africa Countries (Table 3).

## Discussion

The overall aim of this study was to investigate the pooled prevalence and determinates of skilled birth attendant delivery in the 12 East Africa Countries from 2008 to 2017 using recent Demographic and Health Surveys dataset. The pooled prevalence of skilled birth attendants in East African countries was 67.18% (95% CI: 66.98, 67.38) with the highest skilled birth attendant in Rwanda (90.68%) and the lowest skilled birth attendant in Tanzania (11.91%). This was lower than studies conducted in Namibia 80.3% [28]. The finding was much higher than studies conducted in Cambodia 19.8% [17], Bangladesh 35.9% [22], sub-Saharan African countries 53% [15], Ethiopia 28.6% [29], Togo 66.67% [19], and Nepal 48% [30]. The discrepancy might be due to differences in the study period that is Namibia study use DHS 2006/07 while this study 2008\2017. Studies in Cambodia, Bangladesh, Ethiopia, Togo, and Nepal are not representative of the East Africa region since a single country study. The study conducted sub-Saharan Africa include included 34 countries, cultural barriers towards utilization of maternal health services, and different infrastructure across the regions [31].

In East Africa, skilled birth attendance at birth was significantly varied across counties, ranging from 11.91%% in Tanzania to 90.68% in Rwanda. Since 2010, Rwanda has seen a 55% decline in the maternal death ratio. An enormous success that qualified Rwanda to be among the nine developing countries which achieved the millennium development goals 4 and 5 (MDGs). Though skilled birth attendance stands high at 91%, the coverage of completed 4 Ante Natal Care visits remains low at 44% [32]. While in Tanzania according to the Ministry of the health of the country report there is the inadequate implementation of pro-poor policies, weak health infrastructure, limited access to quality health services, inadequate human resources, shortage of skilled health providers [33].

In the multilevel multivariable logistic regression analysis age group, maternal education, husband education, wealth index, antenatal care visit, number of gestation, parity accessing health care, birth interval, residence, and living countries were determinants of skilled birth attendance at birth in the East Africa Countries.

This study evidenced that as the women age group increases the likelihood of obtaining skilled birth attendance at birth also increases. This finding was supported by study findings from Nepal [12] and contradicts studies from Ghana [34]. This could be explained by the fact that as the age of women increases they gained experience and knowledge about the benefit of skilled birth attendants from earlier pregnancies.

Women and her husband who have achieved primary education and higher were more likely to skilled birth attendants than women her husband who didn’t have formal education. This finding is supported by different studies conducted in Vietnam, Togo, Bangladesh, Sub-Saharan Africa, Ethiopia [10, 11, 15, 19, 22]. The finding is also supported by studies in Uganda and Ghana [18, 35]. Attaining education among women and her husband can influence the decision on skilled birth attendance at birth in different ways. Educated women and men would know the benefit of skilled birth attendance at birth and the danger of giving birth by an unskilled professional through reading newspapers, mass media, and from different social media. Overall, educated women and men had good health-seeking behavior and the use of health services [36].

Wealth index had a significant association on the likelihood of skilled birth attendance at birth in the East Africa Countries. This study evidenced that likelihood of obtaining skilled birth attendance at birth was higher among women of middle and rich wealth status as compared to poor women. This finding was supported by studies conducted in Vietnam [11], Togo [19], Bangladesh [13, 22], Ethiopia [29]. The possible justification might be due to women with medium and rich household wealth index were more likely to be able to pay for care-seeking costs such as transportation, medications, and any associated costs and also can get easily information about the benefit of obtaining skilled birth attendance at birth [28].

This study revealed that women who had antennal care visits had a significant association with the likelihood of obtaining skilled birth attendance at birth as compared to women who had no antenatal care visits. This finding was supported by studies Zambia [9], Sierra Leone, Niger, and Mali [16], Ethiopia [10], and Uganda [35]. The possible justification might be due to women during ANC follow up got health education about the advantage of skilled birth attendance at birth. Therefore, women during ANC follow up will got behavioral change towards skilled birth attendance at birth.

The other most significant determinants of skilled birth attendance at birth in this study was the type of gestation. Mothers who have multiple gestations were more likely to obtain skilled birth attendants at birth than singletons. This finding was supported studies conducted from 60 low and middle-income countries [37]. This might be due to the reason that mothers with multiple gestations are at special risk of pregnancy-related complications like obstructed labor, birth asphyxia, antepartum hemorrhage, preeclampsia, and postpartum hemorrhage [38] this could make women seek to obtain skilled birth attendance at birth.

There is a strong relationship between parity and skilled birth attendance at birth. This study evidenced that as the number of children in the family increases the likelihood of obtaining skilled birth attendance at birth decreases. This finding was supported by studies conducted in Nigeria [39], Ethiopia [29], Uganda [35]. The possible justification might be due to multiparous women commonly prefer to give birth at home this results reduction of obtaining skilled birth attendance at birth.

Accessing health care had a significant effect on the likelihood of obtaining skilled birth attendance at birth. The current study evidenced that women who reported accessing health care not big problem increases the likelihood of obtaining skilled birth attendance at birth as compared to women who reported accessing health care big problem. This finding was supported by studies conducted in Ethiopia [10], Ghana [40]. This could be due to the reason that health care access problem is a predisposing factor for seeking skilled birth attendance at birth, and to make maternal health care services available and accessible to the community. This is due to the reason that assessing health care services is important for promoting and maintaining their health, reducing unnecessary disability, and premature death. Besides, accessibility is related to transport issues, financial burden, and long distance to the health facility.

This study evidenced that the odds of obtaining skilled birth attendance at birth among women deliver 2 years and above birth interval were decreased as compared to women deliver less than two-year birth interval. This finding was supported by studies conducted in South East Asia [41]. The possible justification might be due to short birth spacing mothers may face pregnancy complications than long birth spacing which may result in seeking to obtain skilled birth attendance at birth.

Other important determinants of skilled birth attendance at birth in the East Africa counties were residence. The current study evidenced that the odds of obtaining skilled birth attendance at birth among women who reside in the rural area decrease as compared to urban women. This finding was supported by studies conducted in Bangladesh [13, 22], Ghana [42], South Sudan [21], Namibia [28]. The possible justification might be due to there may be high economic concentration, better infrastructure, education, health facilities in urban areas, which may contribute to inequalities in delivery by skilled attendants across all countries in the region.

### Strengths and limitations of the study

Pertaining to the strengths, the dataset used in this study was obtained from nationally representative and the variables in the 12 East Africa DHS dataset were the same hence comparable across all countries. The study was population-based with a response rate of > 90%. The data were pooled together to create a large sample size and increase the generalizability of skilled birth attendant reported within 5 years preceding each country survey which ranges from 2008 to 2017 and was able to identify the significant determinants of skilled birth attendant across the 12 East African Countries to inform policymakers and planners for their intervention to prioritize.

Regarding the limitations, the finding from this study may not establish a true causal relationship between the outcome variable and independent variables due to the cross-sectional nature of the study design. The data was collected based on self-report from mothers within 5 years preceding the survey and this could be a potential source of recall and misclassification bias. Six countries had no DHS data in East Africa and the result of this study may not representative of the entire East Africa country.

## Conclusions

Skilled birth attendance at birth in the East Africa countries was low. Age, women and husband education, wealth index, ANC visit, number of gestation, accessing health care, residence, and living countries were major determinants of skilled birth attendance. Strategies to increase the accessibility and availability of healthcare services, and financial support that enables mothers from poor households to use health services will be beneficial. Health education targeting mothers and their partner with no education are vital to increasing their awareness about the importance of skilled birth attendance at birth.

## Data Availability

Data is available online and you can access it from www.measuredhs.com.

## Abbreviations

ANC: Antenatal Care
AOR: Adjusted Odds Ratio
CI: Confidence Interval
DHS: Demographic Health Survey
ICC: Intra-class Correlation Coefficient
LLR: Log-likelihood Ratio
LR: Likelihood Ratio
MOR: Median Odds Ratio
SBA: Skilled Birth Attendance
SSA: Sub-Saharan Africa
WHO: World Health Organization
